# Health Tract.—No. 60

**Published:** 1865

**Authors:** 


HEALTH TRACT.—No. 60.

### TAKING MEDICINE.

LET it be remembered that it is not the medicine advised by the educated physician which has done the world so much injury, but it is the physic which the people swallow on their own responsibility. When a narrow-minded person gets sick, he "calculates" the saving it will be to him to give twenty-five cents for a box of pills, instead of "employing a physician," besides avoiding the discomfort of "a course of medicine," as it is called. This answers for a while in many cases, but it is ultimately disastrous, and health and life are the fearful forfeit. A gentleman had been a dyspeptic, and hearing that a preparation of soda was "good for dyspepsia," he "tried it;" it acted "like a charm," and for the next six months he was so enraptured with its effects that he considered it a duty as well as a humanity to recommend it to every person who seemed to be affected as he had been. Not long thereafter, as he was standing at the gate of his newly-married daughter, in London, in a passing call on his way to business, he dropped down dead. On examination, the cause was found in several ounces of soda impacted in the bowels.

Not long ago, a young lady of wealth called for a prescription at a Quaker druggist's. Being a conscientious man, he said to her very kindly that if she continued to take it in such quantities, it would destroy her. It was a preparation of morphine, chloroform, and ether, which had an instantaneous and powerful effect on the whole system, and in her case excited the brain and kept it in that condition, requiring constantly increased doses. Within a month she was attacked with a very familiar disease, cured every day in its more peculiar seat. In her case, the brain having been so weakened by the continual over-excitement to which it had been subjected, became the point of metastasis. In familiar phrase, "it went to the brain." She was a model of unobtrusive, self-denying piety, so retiring, so pure, as to be the admiration of those who knew her inner life. In an hour the malady made a wreck of the mind. No man could hold her. Her profanity was shocking to every attendant. A day or two more and she died. We personally know that her sister perished a year earlier in consequence of a condition of the system induced by taking daily, for months a popular "cough-lozenge," or "troché." In these last two cases, economy was no object, for they had always been the pampered and petted children of lavish wealth. But it was so much easier to get rid of an ailment in this way than by the formality of calling in the family physician; besides parental solicitudes need not be uselessly excited; this, no doubt, was the ruling motive. The experienced practitioner well understands that the habitual takng of any efficient medicine is the certain road to a premature and very often a violent or agonizing death

